# Influence of Reinforcement Structures and Hybrid Types on Inter-Laminar Shear Performance of Carbon-Glass Hybrid Fibers/Bismaleimide Composites under Long-Term Thermo-Oxidative Aging

**DOI:** 10.3390/polym11081288

**Published:** 2019-08-01

**Authors:** Juanzi Li, Wei Fan, Yanli Ma, Lili Xue, Linjia Yuan, Wensheng Dang, Jiaguang Meng

**Affiliations:** School of Textile Science and Engineering, Xi’an Polytechnic University, Xi’an 710048, China

**Keywords:** reinforcement structures, 3D orthogonal woven composites, thermo-oxidative aging, inter-laminar shear performance

## Abstract

The effects of reinforcement structures and hybrid types on the inter-laminar shear strength (ILSS) of carbon-glass hybrid fibers/bismaleimide composites under thermo-oxidative aging conditions were investigated. The process resulted in progressive deterioration of the matrix and fiber/matrix interfaces, in the form of chain scissions, weight loss, and fiber/matrix debonding, which significantly led to the decrease of the ILSS of composites. Moreover, the three-dimensional orthogonal woven hybrid composites (3D composites) showed higher ILSS retention rate than those of the laminated orthogonal hybrid composites (laminated composites). No delamination occurred in the aged 3D composites like in the aged laminated composites. This was because the Z-binder yarns in the 3D composites resisted the inter-laminar shear load, although the resin was damaged and the adhesive force between fiber bundles and resin decreased seriously after thermo-oxidative aging. Meanwhile, the ILSS retention rate of the laminated composites with the carbon fiber as intermediate layers was higher than that of the laminated composites with the glass fiber as the intermediate layers. This was because the carbon fiber/bismaleimide interface bonding performance was stronger than that of the glass fiber/bismaleimide at the same thermo-oxidative aging condition.

## 1. Introduction

Polymer matrix composites (PMCs) used in advanced aerospace applications, such as hypersonic flight vehicles, gas turbines, and spacecraft reentry thermal protection systems [1,2,3], are required to possess extraordinary mechanical properties in harsh environments (temperature, oxidation environment, etc.) [4,5,6]. In particular, carbon fiber (CF)/glass fiber (GF) hybrid composites, with excellent mechanical properties and stealth property, are frequently used in aircraft wing cover [7]. Meanwhile, bismaleimide (BMI) resin matrix composites are currently used as structural materials for both military and commercial aircraft, due to their high specific mechanical strength and stiffness under high service temperature [8,9]. This is especially the case of the future supersonic transport aircraft, which is expected to have a service life of around 80,000 h for a supersonic flight, with a maximum skin temperature of 180 °C [10,11]. At high temperatures (approaching the glass transition temperature (*T_g_*) of the matrix), PMCs are susceptible to oxidative degradation. Although the fibers are stable at such temperatures (close to *T_g_*), the matrix—and especially the fiber/matrix—interface can degrade, which affects the physical and mechanical properties of the aeronautic structure over time [12]. Thus, the influence of thermo-oxidative aging (TOA) on the durability, as well as the reliability and safety, of CF/GF hybrid BMI composite must be better understood and considered in the design.

Akay et al. [13] found that the inter-laminar shear strength (ILSS) of CF/BMI composites deteriorated progressively, and the failure mode of the impact specimens changed from brittle failure in the unaged state to progressive delamination in the aged state. Haque et al. [14,15] examined the changes in flexural properties, along with the changes in microstructures of unidirectional CF/BMI, with [0]_16_ and [90]_16_ specimens after exposure to 260 °C for 3000 h in air. In three-point bending, inter-laminar shear stress leads to delamination preceding the final failure in compressive mode. Wang et al. [16] confirmed that damage cracks initiate in piles of 90° and 45°, then grow through at the fiber/matrix interface and propagate rapidly between different oriented piles, leading to the delamination failure of multidirectional CF/BMI composites aged at 200 °C in air for up to 1000 h. Lv et al. [17] found that the CF/BMI composites under flexural loading generated delamination failure after aged at 150 °C. The above researches on the TOA of CF-reinforced BMI composites indicated that the specimens would show delamination damage, whether it suffered bending stress or impact stress in the aged state. This was because of mismatch of thermal expansion coefficients of fiber and resin [18], as well as the resin shrinkage [19], causing fiber/matrix interface debonding during the TOA process [12,18]. The layers in the laminated PMCs are connected only by resin, so once the adhesive force between fiber bundles and resin seriously decreases after TOA, the delamination damage easily occurs with the external load. By contrast, three-dimensional (3D) orthogonal composites can offer increased resistance to delamination due to the addition of Z-binder yarns [20,21]. Hence, it is necessary to investigate the effects of reinforcement structures on the mechanical properties of CF/GF hybrid BMI composite under the TOA condition.

Brian [22] pointed out that the CF/GF hybrid epoxy composites were easily damaged at the place where the hybrid fibers touched each other under TOA condition. It indicated that the hybrid effect might exist in the hybrid composite when it suffered TOA condition. Besides, Bin Yang [23] found that the bonding status between CF/epoxy was better than that of GF/epoxy specimens. This means that the hybrid types of the fibers in the hybrid composite might affect the final mechanical properties under TOA condition. However, limited experimental and analytical studies have been conducted to evaluate the behavior of hybrid PMCs with different reinforcement structures after long-term TOA.

In light of the preceding discussion, the aim of this study was to investigate the effects of reinforcement structures and hybrid types on the inter-laminar shear strength (ILSS) of carbon-glass hybrid fibers/bismaleimide composites under long-term TOA conditions. Fourier transform infrared (FTIR) spectroscopy was employed to determine the influence of long-term TOA on the chemical structures of the matrix. The double-notch shear test was performed to investigate the ILSS of composites. The fracture morphologies of all composites were observed from the macroscopic and microscopic perspectives.

## 2. Experimental Procedure

### 2.1. Materials

Commercially available carbon fibers (T800-12K-CF) and two types of glass fibers (S-12K-GF and S-6K-GF), were used for this study. BMI resin was used for the matrix. The curing process of the BMI resin was 180 °C for 3 h and 230 °C for 3 h.

**Figure 1:**
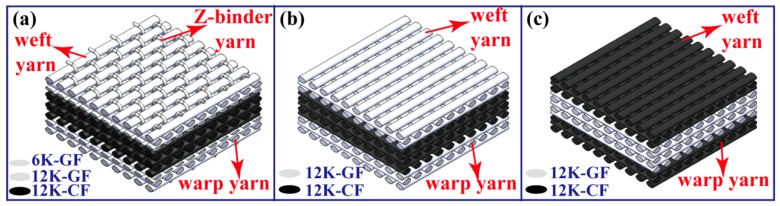
Schematic diagrams of (**a**) the 3D orthogonal woven hybrid preform (1#), (**b**) the laminated orthogonal hybrid preform (2#), and (**c**) the laminated orthogonal hybrid preform (3#).

The 3D orthogonal woven hybrid preform (1#) is shown in Figure 1a. The first four and last four layers used S-GF, and the intermediate five layers used T800-12K-CF. The Z-binder yarns used S-6K-GF. The laminated orthogonal hybrid preforms, which were made by 11 piles of unidirectional fiber cloth, were divided into two types: One is defined as 2# (Figure 1b), in which the first three and last three layers used S-12K-GF and the intermediate five layers used T800-12K-CF; and the other is defined as 3# (Figure 1c), in which the first three and last three layers used T800-12K-CF and the intermediate five layers used S-12K-GF. The performed parameters of the 3D orthogonal woven hybrid preform and the laminated orthogonal hybrid preform are shown in Table 1.

**Figure 2:**
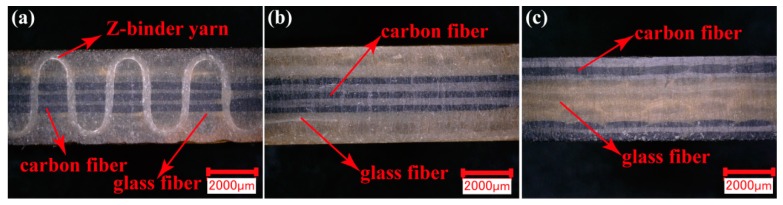
The cross-section microscope pictures of composites: (**a**) The 3D orthogonal woven hybrid composite (Com 1#), (**b**) the laminated orthogonal hybrid composite (Com 2#), and (**c**) the laminated orthogonal hybrid composite (Com 3#).

The Com 1#, Com 2#, and Com 3# referenced by the preform of 1#, 2#, and 3#, respectively, were manufactured by the vacuum assisted resin transfer molding (VARTM) technique—the making process detailed in our previous work [24]. The size of the composite is 25 × 18.5 × 4 mm^3^. The cross-section microscope pictures of the Com 1#, the Com 2#, and the Com 3# are shown in Figure 2, and the profile of the Z-binder yarn in the 3D woven PMCs is shown clearly in Figure 2a.

### 2.2. Thermo-Oxidative Aging

**Figure 3:**
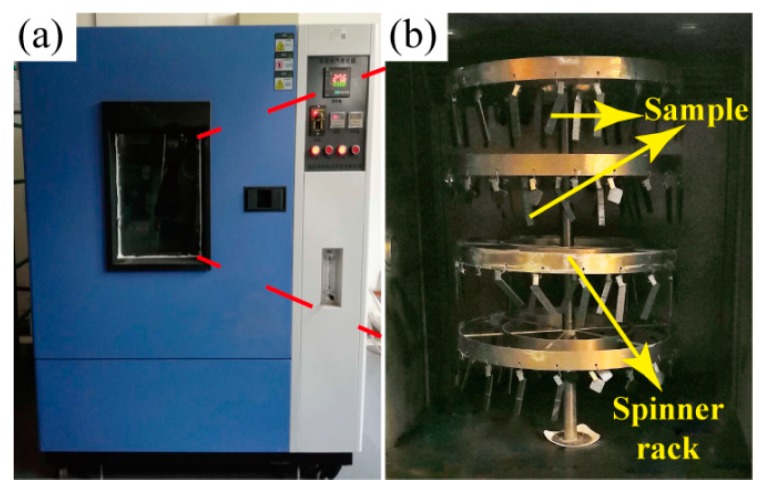
The air-circulating oven: (**a**) The outer of the air-circulating oven, and (**b**) the state of samples in the air-circulating oven.

Specimens for the ILSS measurements were dried in a vacuum oven at 70 °C until a constant weight was achieved, before aging in the air-circulating oven at 250 °C for various periods up to 180 days. It should be noted that the *T_g_* of BMI resin was 230 °C (supplied by the manufacturer, Jiangsu Hengshen Co., Ltd, Jiangsu, China). The 250 °C was selected as the aging temperature in order to accelerate the aging process of the composites. The detailed state of samples in the air-circulating oven is shown in Figure 3. After removal from the oven, the specimens were allowed to cool to room temperature in a desiccator.

### 2.3. Test Methods

#### 2.3.1. Fourier Transform Infrared (FTIR) Spectroscopy

In order to study the influence of TOA on the chemical structures of the BMI resin, FTIR spectroscopy was performed using Spotlight 400 with Attenuated Total Reflectance (ATR) mode. Com 2# specimen was selected to study the changes in chemical structures of the BMI resin.

#### 2.3.2. Mass Loss

The mass loss of the aged specimens was periodically measured by the Sartorius electric weight scale with an accuracy of 10^−5^ g. The mass variation results shown in the figure were the average values of the three samples. Mass loss rate *M_Loss_* was calculated by the following Equation (1) [25]:

(1) $$M_{Loss}\;=\;\frac{M_{0}-M_{t}}{M_{0}}\times 100\%$$

where *M*_0_ is the weight of the original specimen and *M_t_* is the weight of aged specimen at time *t*.

#### 2.3.3. Inter-Laminar Shear Strength Testing

A previous study showed that complex fracture—rather than the simple inter-laminar shear failure—was prone to occur in the short-beam shear test [26]. However, the unidirectional graphite/epoxy always fractured in a single plane under the double-notch shear test [27]. Therefore, we chose the double-notch shear test method to investigate the inter-laminar shear behaviors of the 3D orthogonal woven composites and the laminated composite, before and after TOA. Specimens with the length of 79.5 mm, the width of 12.7 mm, the thickness of 4 mm, and notch depth of 2 mm (the two notches were 6.4 mm apart) were prepared (Figure S1; see the Supplementary Materials) according to ASTM D3846-02. The ILSS *τ* is calculated using:

(2) $$\tau \;=\;\frac{P}{WS}$$

where *P* is the applied load, *W* is the specimen width, and *S* is the spacing between two notches.

#### 2.3.4. Morphology Characterization

The fracture morphologies of specimens were observed by the VHX-5000 Ultra-field microscopy system. The fiber/matrix interface morphologies were observed using the scanning electron microscope (SEM, Quanta-450-FEG, FEI Co., Hillsborough, CA, USA).

## 3. Results and Discussion

### 3.1. Effects of TOA on Chemical Structures of BMI Resin

**Figure 4:**
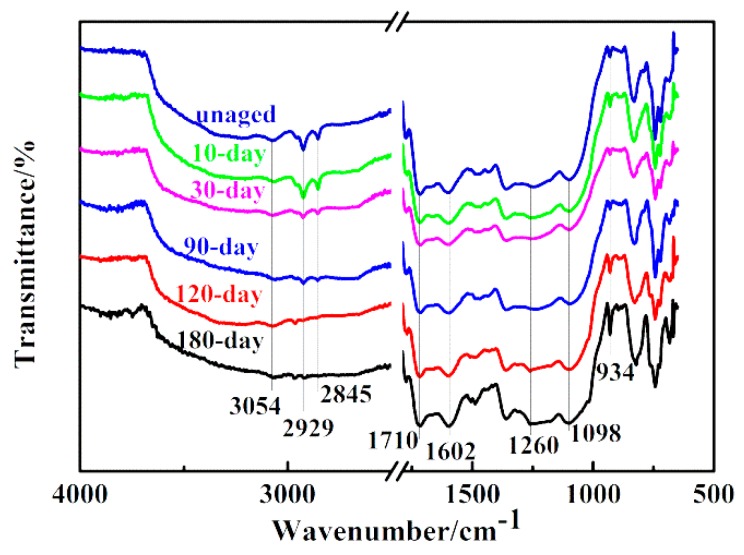
Fourier Transform Infrared (FTIR) spectra of the bismaleimide (BMI) composite aging for different periods.

FTIR spectra (Figure 4) present the changes in chemical structures of the BMI resin for various thermal aging periods, and Table 2 summarizes the characteristic peaks of the aged BMI resin. Figure 4 shows that the absorption at 934 cm^−1^ increases obviously when composites suffer long-term TOA (120 days and 180 days), which indicates that more and more cross-linked maleimide ring decomposed. The increasing absorption at 1710 cm^−1^ is believed to be associated with such double bonds caused by TOA. Additionally, the absorption at 1602 cm^−1^ becomes stronger and stronger from 0 to 180 days, which confirms that the methylene bridges (–CH_2_–) in cured BMI resin are oxidized and formed C=O conjugated to a benzene ring. While the sharp declining peaks at 2929 cm^−1^ and 2845 cm^−1^ confirm that susceptible hydrocarbon units are heavily oxidized after long-term TOA. The change at 3054 cm^−1^ (bonded –OH) indicates that matrix suffered strong oxidative degradation. Furthermore, the changes at both 1260 cm^−1^ and 1098 cm^−1^ are also related to decomposition. Consequently, the changes in chemical structures of matrix resin are attributed to the decomposition of cross-linked maleimide after aging for different time at 250 °C, which results in the deterioration of BMI resin.

### 3.2. Effects of TOA on Mass Loss

**Figure 5:**
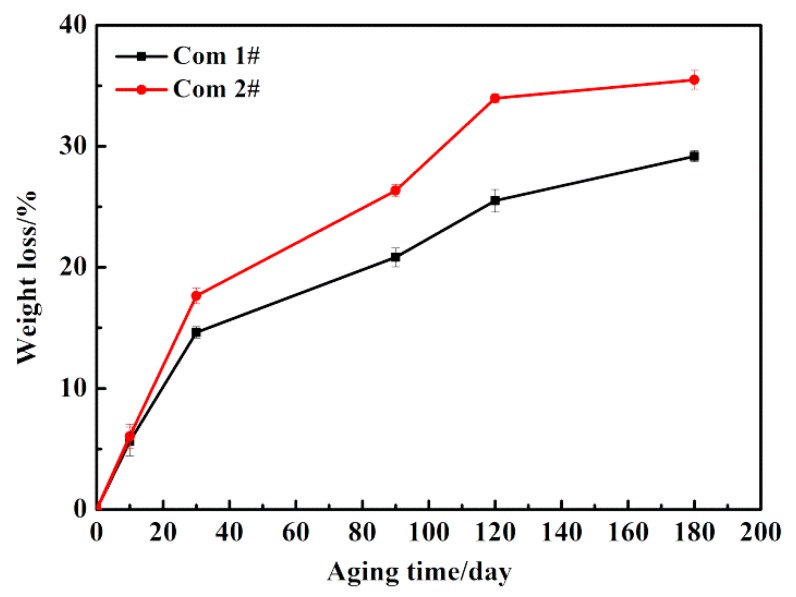
Weight loss of the Com 1# and Com 2# vs. aging time.

Figure 5 shows the weight loss of the Com 1# and the Com 2# with different aging time. It was found that the weight loss of the two materials increased with longer aging time. The Com 1# and the Com 2# lost 29.18% and 35.48% of their original weights, respectively. This is owing to the oxidative decomposition, as illustrated in Figure 4. In addition, there is a remarkable variation in mass loss between the Com 1# and the Com 2#. This is mainly because the fiber volume fraction of the Com 1# (57.24%) is bigger than the Com 2# (55.26%), which means that there is much matrix resin content in Com 2# than in Com 1#. The weight loss of CF/GF PMCs comes from the matrix resin rather than the fibers [14,30,31], so the weight loss of the Com 2# is bigger than that of the Com 2#. There might also exist a possible reason for this difference is that the excellent structural integrity of the Com 1#, which hinders the motion of molecular chain effectively.

### 3.3. Effects of Hybrid Structures on the Inter-Laminar Shear Property

**Figure 6:**
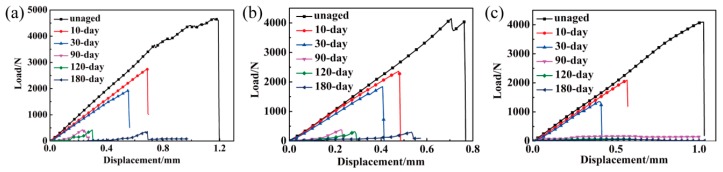
The load-displacement curves for (**a**) the Com 1#, (**b**) the Com 2#, and (**c**) Com 3# at 250 °C aged for different periods.

The typical load-displacement curves of the three composites are presented in Figure 6. As seen in the figure, loads of all composites decrease as the thermal aging time increases. For all the composites, the load of unaged composites and composites aged for 10 days and 30 days increase sharply at the initial stage, and catastrophic failure occurs immediately when they reach the peak value. While the initial stage of both the Com 1# and Com 2# that aged for 90 days and 120 days increase slowly, this indicates that plastic damage occurred in the composites resulted from the degradation of BMI matrix after long-term TOA [32].

**Figure 7:**
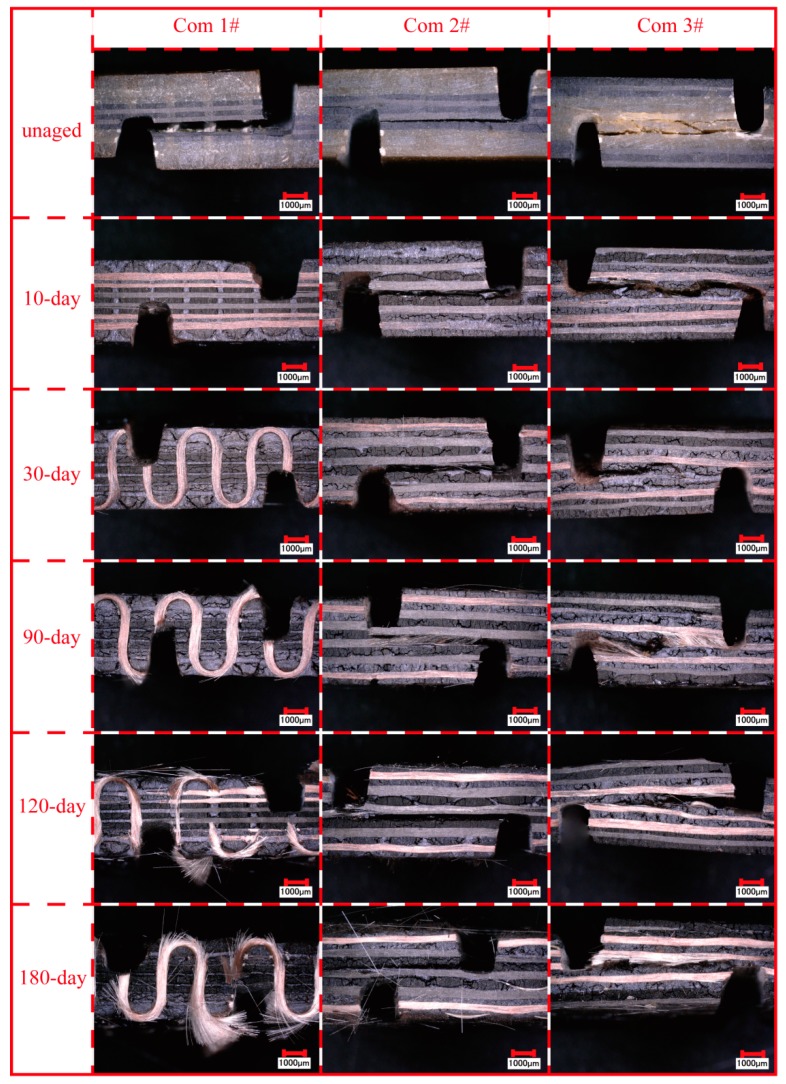
The fracture morphologies of unaged and aged inter-laminar shear specimens.

Figure 7 shows microscopic images of unaged and aged specimens after the inter-laminar shear test. The unaged specimens only show significant internal delamination at intermediate piles. In contrast, aged specimens show much looser fiber bundles, resulting from the deteriorated matrix caused by long-term TOA. Additionally, longitudinal and transverse microcracks are observed obviously in aged specimens that provided pathways for further oxidation in the inner core. The microcracks between fiber tows and resin are due to the different thermal stress in fibers and resins during the long-term TOA [18]. The coefficients of thermal expansion (CTE) of carbon fiber and glass fiber are −0.38 × 10^−6^/°C and 2.59 × 10^−6^/°C, respectively (provided by the manufacturer). The CTE of BMI resin is 44 × 10^−6^/°C [3]. Hence, the mismatch in the CTE between the fibers and resin gives rise to localized thermal stresses at the fiber/matrix interface, which makes it prone to microcracks. The increase in microcrack density is due to the deterioration of the matrix resin after exposure to long-term TOA (as illustrated in Figure 4), which promotes degradation in the fiber/matrix interface leading to obvious delamination. Comparing the aged Com 1# with the aged laminated orthogonal hybrid composites (the Com 2# and the Com 3#), no delamination failure mode occurs in the former, while the latter all suffered delamination failure after long-term TOA. Moreover, delamination failure occurs directly at the two notch locations in the two laminated orthogonal hybrid composites of three aged for 180 days before the inter-laminar shear test (as shown in Figure 7). This behavior indicates that the structural integrity of the laminated orthogonal hybrid composites is heavily damaged and the composites do not have the load-bearing capacity. However, the aged Com 1# can still bear much load, which is because the Z-binder yarns in the thickness of the composites improve the structural integrity and are helpful in resisting the inter-laminar shear load.

**Figure 8:**
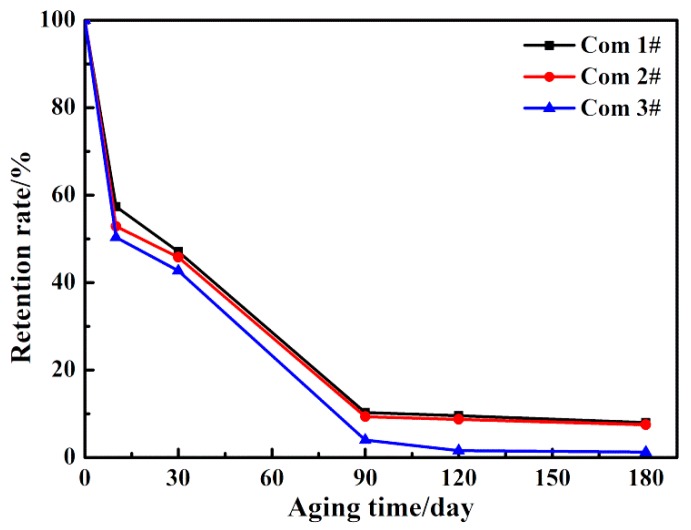
The inter-laminar shear strength (ILSS) retention rates of the three composites at 250 °C for different aging periods.

The ILSS retention rate of the three composites is presented in Figure 8, and the ILSS values of the three composites are shown in Table S1. Notably, the ILSS value and the ILSS retention rate of all specimens decrease with the increasing thermal aging time, which resulted from the deterioration of the matrix resins and fiber/matrix interface caused by long-term TOA. Furthermore, the Com 1# show higher ILSS values and ILSS retention rates than the laminated orthogonal hybrid composites. This is because the Z-binder yarns in the Com 1# improve the structural integrity and can resist the inter-laminar shear load effectively when the resin is damaged and the adhesion of fiber/matrix interface decreases severely after long-term TOA.

### 3.4. Effects of Hybrid Types on Inter-Laminar Shear Properties

In Figure 6, compared with the Com 2#, the Com 3# completely loses its load-bearing capacity after aging for more than 90 days. Meanwhile, as shown in Figure 8, the ILSS retention rate of the Com 2# is higher than that of the Com 3#.

**Figure 9:**
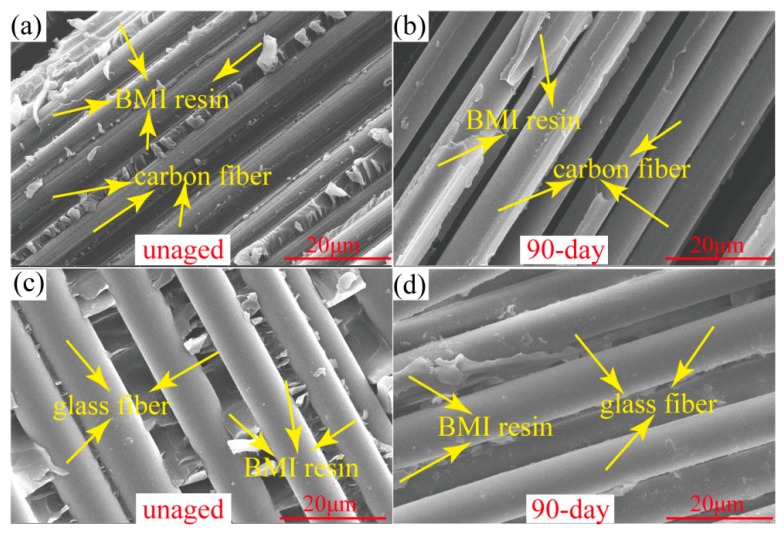
Scanning electron (SEM) pictures of fiber/matrix interface: (**a**) The unaged Com 2#, (**b**) the Com 2# at 250 °C aged for 90 days, (**c**) the unaged Com 3#, and (**d**) the Com 3# at 250 °C aged for 90 days.

To further understand the influence of the hybrid types on the inter-laminar shear property, the SEM morphologies of CFs/matrix and GFs/matrix interfaces in unaged composite and composite aged for 90 days after the inter-laminar test are shown in Figure 9. It is noted that the fibers of unaged composite are covered with matrix (Figure 9a,c), which indicates the good interfacial adhesion. After aging for 90 days, there is less resin adhered to the fibers’ surface and severe interface debonding occurs between the fibers and matrix, as shown in Figure 9b,d. Qualitatively, with the aging time increasing, the amount of matrix left on the surface of the hybrid composites decreases, which causes more fibers to become exposed (as shown in Figure 7). Both the outer matrix degradation and inner interface oxidation lead to the continuous reduction of ILSS, as shown in Figure 6. Moreover, the carbon fiber-bundle surfaces have massive residual BMI resin after the inter-laminar test, as shown in Figure 9a, while very smooth glass fiber-bundle surfaces are observed in Figure 9c. This phenomenon illustrates that the CFs/BMI interface adhesion is stronger than that of the GFs/BMI. This conclusion is consistent with the mechanical test results in Figure 6, which show that the ILSS of the Com 2# is higher than that of the Com 3# under TOA. This is also confirmed by reference [23].

## 4. Conclusions

The effects of hybrid structures and hybrid types on CF/GF/BMI hybrid composites under TOA condition are discussed in the present study. ILSS of the Com 1#, the Com 2#, and the Com 3# aged at 250 °C for various periods up to 180 days were examined. The results obtained are given here briefly:

1. Long-term TOA resulted in progressive deterioration of the matrix resins and fiber/matrix interfaces, in the form of chain scissions, weight loss, and fiber/matrix debonding, which led to the dramatic decrease of ILSS of hybrid composites.
2. The Com 1# showed a higher ILSS retention rate than those of the laminated orthogonal hybrid composites. Also, no delamination occurred in aged Com 1# unlike the aged laminated orthogonal hybrid composites. This was because the Z-binder yarns in the Com 1# can resist the inter-laminar shear load, even under the condition of TOA.
3. The ILSS retention rate of the Com 2# (with the CFs as the intermediate layers) was higher than that of the Com 3# (with the GFs as the intermediate layers). This phenomenon can be explained by the result that the CFs/BMI interface adhesion was stronger than that of the GFs/BMI under the same TOA condition.

## Figures and Tables

**Table 1:** Performed parameters of the 3D orthogonal woven hybrid preform and the laminated orthogonal hybrid preform.

Reinforced Structure	Unit Density/(yarn/cm)			Layers	Fiber Volume Fraction
	Warp	Weft	Z-Binder Yarn		
3D orthogonal woven hybrid preform	5	5	5	13	57.24%
Laminated orthogonal hybrid preform	5	5	-	11	55.26%

**Table 2:** FTIR characteristic peaks position of the aged BMI composite [16,28,29].

Peaks Position (cm^−1^)	Assignment	Functional Group
3054	Water, non-bonded hydroxyl	–OH
2929	Asymmetric CH_2_ stretch	–CH_2_–
2845	Symmetric CH_3_	–CH_3_
1710	Asymmetric imide	–C=O
1602	C=C stretch with C=O conjugation	–C=C–C=O
1260	Various carbon-oxygen	–C–O–
1098	Succinimide or ether	C–N–C, –C–O–C–
934	Maleimide deformation	C=C

## Supplementary Materials

The Supplementary Materials are available online at https://www.mdpi.com/2073-4360/11/8/1288/s1.
