# Supplementary material for: Influence of Reinforcement Structures and Hybrid Types on Inter-Laminar Shear Performance of Carbon-Glass Hybrid Fibers/Bismaleimide Composites under Long-Term Thermo-Oxidative Aging
Source: Polymers (Basel). 2019 Aug 1;11(8):1288. doi: 10.3390/polym11081288 (PMC6723977; doi:10.3390/polym11081288)
Supplement: Supplementary file 1 [file polymers-11-01288-s001.pdf]

Supplementary Caption

Fig. S1 Dimension figure of the double-notch shear sample.

Table S1. The ILSS values of the Com 1#, the Com 2# and the Com 3#.

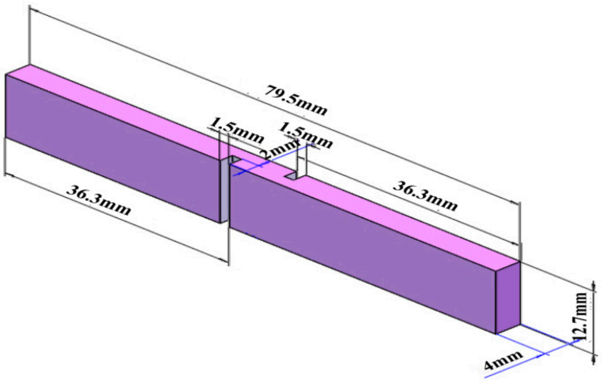

Fig. S1 Dimension figure of the double-notch shear sample.

Table S1. The ILSS values of the Com 1#, the Com 2# and the Com 3#.

samples	Mean ILSS/MPa					
	unaged	10-day	30-day	90-day	120-day	180-day
Com 1#	97.68±16.51	50.51±4.03	41.47±7.56	9.05±1.05	8.43±0.81	7.01±0.63
Com 2#	80.62±6.21	42.63±6.15	36.90±7.81	7.5±0.62	7.01±0.51	6.04[1]
Com 3#	78.10±2.91	39.32±6.45	33.36±4.12	3.11±0.61	1.21±0.19	0.98[1]

6.04[1] and 0.98[1]: Because the delamination failure directly occurred in two laminated orthogonal hybrid composites of three before the inter-laminar shear test, there was only one value of the ILSS of the laminated orthogonal hybrid composites.
